# From Chains to Care: Ending *Pasung* (Physical Restraint) Among Schizophrenia Patients in Indonesia, A Systematic Review

**DOI:** 10.2174/0117450179414516251030074059

**Published:** 2025-11-12

**Authors:** Jesslyn Shi, Rama Mahardika Primanov, Rina Amelia, Nanda Andini

**Affiliations:** 1 Undergraduate Program in Medicine, Faculty of Medicine, Universitas Sumatera Utara, Medan, Indonesia; 2 Department of Community Medicine, Faculty of Medicine, Universitas Sumatera Utara, Medan, Indonesia

**Keywords:** Schizophrenia, *Pasung*, Physical restraint, Mental health, Policy, Stigma, Traditional belief

## Abstract

**Background:**

*Pasung*, the physical restraint and confinement of individuals with mental disorders—particularly schizophrenia—continues to occur in Indonesia, despite the national “Free from *Pasung*” campaign and Mental Health Act No. 18/2014. This systematic review aims to synthesize existing evidence on the prevalence, causes, consequences, and policy responses related to *pasung* among people with schizophrenia in Indonesia.

**Method:**

A systematic search was conducted using PubMed, PsycINFO, Cochrane Library, and the Garuda Indonesian research repository. The quality appraisal were analyzed using tools from the Joanna Briggs Institute (JBI) and CASP. The included studies were analyzed using thematic synthesis guided by the Socio-Ecological Model (SEM).

**Results:**

Eight studies met the inclusion criteria.

**Discussion:**

Findings reveal that *pasung* is driven by persistent stigma, poverty, limited mental health literacy, cultural beliefs attributing illness to supernatural causes, and the lack of accessible, community-based mental health services. The consequences include muscle atrophy, psychological trauma, loss of dignity, and delayed or denied treatment.

**Conclusion:**

The persistence of *pasung* highlights critical gaps in Indonesia’s mental health system, particularly at the community and policy levels. Addressing this issue requires a multi-level approach, including culturally adapted interventions, stronger mental health governance, and community empowerment to support inclusive, rights-based mental health care.

## 1 INTRODUCTION

Schizophrenia is a severe mental disorder characterized by significant impairments in perception, thought, and behaviour, including persistent delusions, hallucinations, and disorganised speech [1]. Globally, it affects approximately 24 million people and is associated with a life expectancy 10–20 years below that of the general population [1, 2]. More than two-thirds of those experiencing psychosis do not receive specialist mental health care, underscoring a substantial treatment gap [1]. In Indonesia, mental health remains significantly under-resourced and stigmatised. In 2023, an estimated 9.16 million adults experienced mental or emotional disorders—about 6.1 per cent of the population—while recorded suicides rose by 36.4 per cent between January and June 2023 compared to the same period in 2021. These figures highlight both the growing burden of mental illness and the inadequacy of current services [3]. Within this context of limited care and pervasive stigma, many families resort to *pasung*, a traditional practice involving physical restraint and confinement of individuals with severe mental illness in community settings. *Pasung* typically employs mechanical restraints—such as chains, shackles, or wooden stocks—and isolation in makeshift cages or locked rooms, often within the person’s home [4]. Although *pasung* was officially banned by a Home Affairs Ministry regulation in 1977 and targeted by the Ministry of Health’s “*Gerakan Bebas Pasung*” (Free from *Pasung*) initiative launched in 2010, followed by the enactment of the Mental Health Act No. 18/2014, its practice persists across many provinces. Fragmented policy implementation, unclear institutional roles, and inadequate accountability mechanisms have limited the effectiveness of these legal measures [5]. Official data from the Indonesian Ministry of Health report a decline in *pasung* cases from 18,880 in 2010 to 12,220 in 2018, yet these figures likely underestimate true prevalence due to familial concealment and inconsistent reporting across districts. Discrepancies between government claims and independent surveys further cast doubt on the accuracy of these estimates [5]. The continued use of *pasung* is driven by a complex interplay of factors: deep-rooted cultural beliefs attributing mental illness to supernatural causes, pervasive social stigma, low levels of mental health literacy, and a lack of accessible community-based services, especially in rural regions [4, 5]. Families often perceive *pasung* as the only available strategy to manage perceived risks posed by affected relatives [4]. Prolonged confinement under *pasung* inflicts severe physical and psychological harm. Reports document undernutrition, untreated comorbid medical conditions, injuries from restraints, exacerbation of psychiatric symptoms, and long-term trauma, sometimes culminating in preventable morbidity and mortality [4]. These outcomes reflect gross violations of human rights and underscore the urgency of addressing *pasung*. Despite recognition of its detrimental impacts, research on pasung remains scarce and methodologically limited. Few studies have rigorously evaluated interventions, and existing qualitative work highlights the need for integrated, culturally sensitive solutions [4]. Emerging empirical evidence also questions government-reported declines in *pasung* cases and calls for more robust surveillance [6]. Consequently, a systematic review is warranted to synthesise recent findings on the prevalence, drivers, harms, and interventions related to *pasung* among schizophrenia patients in Indonesia [3, 4, 6]. This is the first systematic review to discuss the use of *pasung* among patients with schizophrenia in Indonesia.

## 2 METHODS

The protocol for this systematic review was developed and subsequently registered with PROSPERO (ID: 1061376). Upon acceptance of the registration, the systematic review process commenced with a comprehensive literature search, followed by screening of identified studies, and culminating in the extraction of data from eligible papers.

### 2.1 Literature Searching

To identify relevant primary studies, a comprehensive literature search was carried out across several academic databases, including PubMed, PsycINFO, the Cochrane Library, and Garuda, which is an Indonesian research repository. We performed a comprehensive literature search covering the period from January 1, 2025, to April 1, 2025. The following search string was used: “Schizophrenia” AND “Pasung” OR “Physical Restraint” AND “Indonesia.” Below are the inclusion and exclusion criteria. The inclusion criteria included studies focusing on *pasung* among schizophrenia patients in Indonesia; quantitative, qualitative, and mixed-methods studies; articles published in English or Bahasa Indonesia; peer-reviewed journal articles, government reports, and NGO publications; and studies published no more than 15 years ago. The exclusion criteria included: studies focusing on *pasung* outside Indonesia; systematic reviews and meta-analyses; studies that do not specify schizophrenia as the primary condition; non-peer-reviewed opinion articles or non-relevant case reports.

### 2.2 Screening Process

Initially, the authors independently screened the titles and abstracts of all retrieved articles based on the predetermined inclusion criteria. Each article was categorized as *“included,” “excluded,”* or *“uncertain.”* Following this initial screening, both reviewers discussed any discrepancies in the *“included”* and *“excluded”* categories to reach consensus. For articles classified as *“uncertain,”* further deliberation was conducted, and in cases where agreement could not be reached, RA served as a third-party adjudicator to resolve any remaining disagreements. Subsequently, a full-text review of all potentially eligible studies was conducted by JS and RMP to determine whether they met the inclusion criteria in full. RA again acted as a mediator if uncertainties or disagreements persisted during this stage. Risk of bias assessments for each included study were then performed by JS, RMP, RA, and NA using appropriate critical appraisal tools such as CASP, JBI, or others, depending on the study design. For articles published in Bahasa Indonesia, JS and RMP, native speakers of Bahasa Indonesia, conducted a thorough screening, followed by an in-depth discussion with RA and NA to ensure accurate interpretation and inclusion decisions. Final agreement on the list of included studies was reached through comprehensive discussion among all authors to ensure consensus and methodological rigor.

### 2.3 Data Extraction and Analysis

The data extraction process was primarily conducted by the first author and subsequently reviewed for accuracy and completeness by all members of the research team. A standardized data extraction form was used to collect study characteristics (year, location, study design, sample size) and key findings on prevalence, risk factors, consequences, interventions, and limitations of each study.

### 2.4 Quality Assessment

Only the included literature underwent quality assessment. We utilized JBI tools for qualitative studies and CASP for cohort studies. However, the quality assessment was used only to further evaluate the value of each study, not to eliminate any study. Due to the limited number of eligible studies identified following the full-text screening, the authors decided to include all available literature regardless of methodological quality, in order to provide a comprehensive overview of the topic. All authors personally reviewed each study, and further discussion was held to finalize the rating for each study.

To guide thematic synthesis, we employed the Socio-Ecological Model (SEM) as a conceptual framework to organize and interpret findings across multiple levels of influence. Initially, data were coded inductively to allow themes to emerge from the included studies without imposing predefined categories. After open coding, emergent themes were subsequently mapped onto the five levels of the SEM: individual, interpersonal, community, institutional (service/system), and policy. This iterative process allowed us to capture the complexity of the factors sustaining *pasung* practices, while ensuring consistency with an established theoretical structure. Themes that did not fit neatly into a single level were discussed and categorized based on their predominant context. This approach enabled us to contextualize the drivers, consequences, and policy gaps surrounding *pasung* within a multi-layered analytical lens.

In addition to peer-reviewed articles, this review incorporated selected gray literature sources, such as reports from Non-Governmental Organizations (NGOs), government documents, and policy briefs, to capture a broader range of data relevant to pasung practices. We critically assessed each source for transparency, consistency with peer-reviewed findings, and potential bias. While we acknowledge that gray literature may vary in methodological rigor, it provided valuable context on real-world implementation and policy narratives that are often underreported in academic literature.

## 3 RESULTS

### 3.1 DATA CHARACTERISTICS

**Fig. (1):**
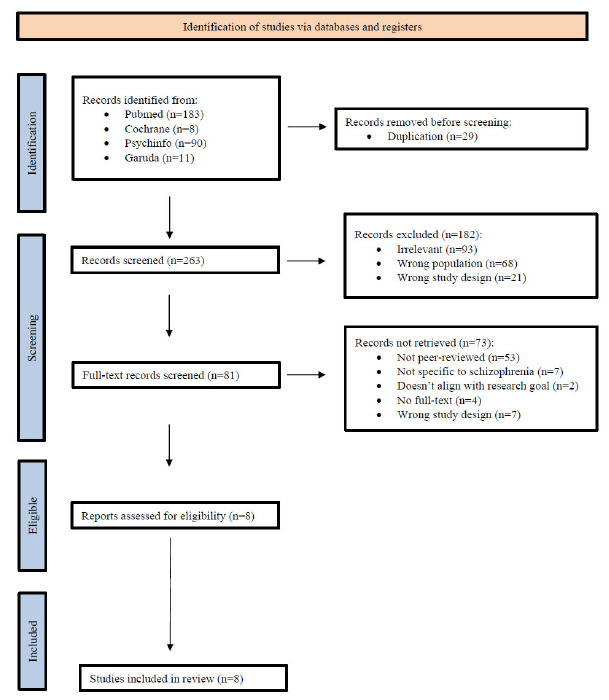
PRISMA Flow Diagram (journal articles inclusion flow chart).

Figure. **1** shows the PRISMA flow diagram of this study. A total of 292 records were identified through database searching, comprising 183 from PubMed, 90 from PsycINFO, 8 from Cochrane, and 11 from Garuda, an Indonesian local journal. Following the removal of 29 duplicate records, 263 records proceeded to the screening stage. During this phase, 181 records were excluded due to irrelevance (n = 93), wrong population (n = 68), and inappropriate study design (n = 21). Consequently, 81 full-text articles were assessed for eligibility.

Out of the 81 full-text articles, 73 were excluded due to various reasons: 53 were not peer-reviewed, 7 were not specific to schizophrenia, 7 employed an unsuitable study design, 4 had no accessible full-text, and 2 did not align with the research goals. This led to a total of 8 studies being assessed and deemed eligible, all of which were subsequently included in the final review. No additional reports were excluded at this stage. Overall, the selection process demonstrates a rigorous and transparent screening method, ensuring that only high-quality and relevant studies were incorporated into the review. All of the 8 articles that were included in this review were written by researchers in Indonesia and collaborated with researchers from the Netherlands, Australia, England, and Japan. Of the 8 papers, 3 were quantitative (cross-sectional and correlational), 3 were qualitative (grounded theory and deep interview), 1 was a policy analysis, and 1 was a program implementation paper.

Given the methodological heterogeneity of the included studies, a meta-analysis was not feasible. The data sources consisted of diverse study designs, many of which lacked standardized outcomes or quantifiable effect estimates. Additionally, variations in definitions, participant populations, and regional contexts further impeded statistical synthesis. Therefore, we conducted a systematic review to thematically integrate the findings and highlight common patterns across settings.

### 3.2 Case Control Study

One case-control study was analyzed using the JBI Critical Appraisal Checklist for Case-Control Studies, which contains 10 questions. Of 10 questions, only 7 were answered with “Yes”. These questions are: Question 1, “Were the groups comparable other than the presence of disease in cases or the absence of disease in controls?”—This case-control study met this criterion. Question 2, “Were cases and controls matched appropriately?”—This study does not have any controls. Question 3, “Were the same criteria used for identification of cases and controls?”—This study uses the same diagnostic criteria to assess the cases and controls, despite this study not using any control group. Question 4, “Was exposure measured in a standard, valid, and reliable way?”. Question 5, “Was exposure measured in the same way for cases and controls?”. Exposure assessment used in this study and utilized standardized instruments with established validity, using semi-structured questionnaires and corroborated by health-service medical records. Question 6, “Were confounding factors identified?”—The study acknowledged potential confounders such as socioeconomic status and family support. Question 7, “Were strategies to deal with confounding factors stated?” Multivariate analyses were conducted to adjust for identified confounders. Question 8, “Were outcomes assessed in a standard, valid, and reliable way for cases and controls?”—The study did not detail the validation of outcome assessment tools, raising concerns about reliability. Question 9, “Was the exposure period of interest long enough to be meaningful?”—The study lacked information on the duration of exposure assessment, limiting temporal relevance. Lastly, Question 10, “Was appropriate statistical analysis used?”—Statistical methods were suitable for the study design and data type. Based on the JBI appraisal tool, this study was categorized as having a moderate risk of bias.

### 3.3 Cross-Sectional Study

Two additional studies assessed in this review included a descriptive and analytic cross-sectional study. The JBI Critical Appraisal Checklist for Studies Reporting Prevalence Data and the JBI Critical Appraisal Checklist for Analytical Cross-sectional Studies were used for their evaluation.

The JBI tool for descriptive study has 9 questions. Of the 9 Questions, this study answers “Yes” to only 6 questions. The questions are: Question 1, “Was the sample frame appropriate to address the target population?”—The sample frame accurately represented the target population of ex-*pasung* patients. Question 2, “Were study participants sampled in an appropriate way?”—Participants were selected using methods that minimized selection bias. Question 3, “Was the sample size adequate?”—The study did not provide a sample size calculation or justification. Question 4, “Were the study subjects and the setting described in detail?”—Comprehensive descriptions of participants and settings were provided. Question 5, “Was the data analysis conducted with sufficient coverage of the identified sample?”—The analysis did not account for all identified participants, potentially affecting representativeness. Question 6, “Were valid methods used for the identification of the condition?”—Diagnostic criteria for mental illness were appropriately applied. Question 7, “Was the condition measured in a standard, reliable way for all participants?”—Consistent measurement procedures were employed across all subjects. Question 8, “Was there an appropriate statistical analysis?”—Statistical analyses were appropriate for the data collected. Question 9, “Was the response rate adequate, and if not, was the low response rate managed appropriately?”—The study did not address the response rate or strategies to handle non-response bias. Based on the JBI critical appraisal tool, this descriptive cross-sectional study had a moderate risk of bias.

The analytical cross-sectional study uses the JBI Critical Appraisal Checklist for Analytical Cross-Sectional Studies to assess the risk of bias of the study. The tool consists of 8 questions, and this study answered “Yes” to 5 of them. Questions that were used were: Question 1, “Were the criteria for inclusion in the sample clearly defined?”—The study lacked explicit inclusion criteria, which may affect reproducibility. Question 2, “Were the study subjects and the setting described in detail?”—Detailed information about participants and the study setting was provided. Question 3, “Was the exposure measured in a valid and reliable way?”—Exposure assessment utilized validated instruments with reported reliability. Question 4, “Were objective, standard criteria used for measurement of the condition?” Standardized criteria were applied for condition measurement. Question 5, “Were confounding factors identified?”—The study did not identify potential confounding variables. Question 6, “Were strategies to deal with confounding factors stated?—There was no mention of methods to control for confounding. Question 7, “Were the outcomes measured in a valid and reliable way?”—Outcome measures were based on validated tools with established reliability. Question 8, “Was appropriate statistical analysis used?”—Statistical analyses were suitable for the study design and data type. Based on the JBI appraisal tool, this study was concluded as having a moderate risk of bias.

### 3.4 Qualitative Studies

Three studies were conducted using a qualitative approach, and the JBI Critical Appraisal Checklist for Qualitative Studies was used to analyze the risk of bias. The questions are as follows: Question 1, “Is there congruity between the stated philosophical perspective and the research methodology?”—None of the studies articulate specific philosophical perspectives for this research. Question 2, “Is there congruity between the research methodology and the research question or objectives?”—Two of the studies use qualitative study methods, and one of the studies used grounded theory, which is congruent with the research objectives. Question 3, “Is there congruity between the research methodology and the methods used to collect data?”—All of the studies use semi-structured interviews, in-depth interviews, and field notes, and also align with grounded theory’s iterative data collection process. Question 4, “Is there congruity between the research methodology and the representation and analysis of data?”—Two of the studies have content analysis matches the exploratory qualitative methodology and allows valid inference from the sample, as well as the use of Paillé’s grounded theory analytic procedures (open, axial, selective coding) matches the methodology but, one of the study had unclear representation because the authors do not explicitly describe their analytic framework (*e.g.*, thematic analysis, narrative analysis), making congruity between methodology and analysis uncertain. Question 5, “Is there congruity between the research methodology and the interpretation of results?”—All of the study’s interpretation results are congruent with each methodology. Question 6: “Is there a statement locating the researcher culturally or theoretically?”—From all three studies, there is no statement on the researchers’ cultural, theoretical, or personal positioning. Question 7, “Is the influence of the researcher on the research, and vice-versa, addressed?”—The authors from three of the studies do not reflect on how their perspectives or interactions may have shaped data interpretation. Question 8, “Are participants, and their voices, adequately represented?”—Two studies use illustrative quotations throughout the results to anchor themes in participants’ own words, whereas one of the studies remains unclear because it only summarizes key points from the samples. Question 9, “Is the research ethical according to current criteria and is there evidence of ethical approval by an appropriate body?”—All the studies had ethical approval by the ethical approval committee before conducting the study. Question 10, “Do the conclusions drawn in the research report flow from the analysis or interpretation of the data?”—Three studies’ conclusions were consistent with the report flow from the analysis. From the JBI tools, these studies concluded that the risk of bias was moderate.

### 3.5 Policy Analysis

One study reviews the policy about *pasung*, using the JBI Critical Appraisal Checklist for Text and Opinion Papers to assess the risk of bias. This study scores 6 out of 6 questions. Question 1, “Are author(s) and publication details explicit?”—The authors and affiliations of this study are clearly listed. Question 2, “Do authors have relevant expertise or credibility?”—The authors from this study are experienced policy and mental-health researchers. Question 3, “Is the opinion/text centred on the target group?”—Yes, this study examines national and provincial *Pasung* policies. Question 4, “Is there a coherent, systematic argument or framework?”—This study uses the Policy Triangle Framework and content analysis. Question 5, “Does the paper engage with existing evidence or theory?”—The paper uses 83 refs spanning policy and empirical studies. Question 6, “Are any conflicts with existing sources or literature logically addressed?”—This research identifies ambiguous messaging and decentralization gaps. Overall, this study was classified as low risk of bias from the JBI tool.

### 3.6 Program Implementation

Another paper describes a program implementation, using the same JBI Critical Appraisal Checklist for Text and Opinion Papers tool to assess the risk of bias. This study also answered 6 out of 6 questions. The questions are: Question 1, “Are author(s) and publication details explicit?”—The authors and affiliations of this study are clearly stated. Question 2, “Do authors have relevant expertise or credibility?”—The authors from this study from Mind Australia, Univ. Melbourne, St Vincent’s. Question 3, “Is the opinion/text centred on the target group?”—Yes, it targets the homeless/*pasung*-affected mentally ill individuals in Sukabumi. Question 4, “Is there a coherent, systematic argument or framework?”—This paper describes programme design, implementation, and lessons learned. Question 5, “Does the paper engage with existing evidence or theory?”—The paper uses references, recovery frameworks, and prior studies. Question 6, “Are any conflicts with existing sources or literature logically addressed?”—This research discusses contextual challenges and adaptations. This study was categorized as low risk of bias from the JBI tool.

### 3.7 Key Findings

The key findings of the included studies in this systematic review are shown in Table **1**.

### 3.8 Prevalence of *Pasung* in Schizophrenia Patients

The prevalence of *pasung* in schizophrenia patients in Indonesia varies, but it is notably higher in rural areas compared to urban areas. Several studies report that *pasung* is still prevalent, especially in rural areas, despite national efforts to reduce it [5, 7, 10]. In a study, family members such as fathers, mothers, and elder siblings are those who initiate *Pasung* [7]. Movements like *“Aceh Free Pasung”* discuss regional efforts to eliminate *pasung* in Aceh but acknowledge challenges in more remote areas. Although prevalence data were not always reported numerically, studies suggest that *pasung* remains common in rural communities [9].

### 3.9 Factors Leading to the Practice of *Pasung*

The Socio-Ecological Model (SEM) was used to analyze the factors leading to *pasung*. These factors were categorized into individual, family, community, and policy levels, using thematic analysis to group them according to recurring themes. However, no studies in this review directly explored the perspectives, experiences, or beliefs of individuals with schizophrenia themselves. All data at this level were reported indirectly, mainly from family or community viewpoints. Therefore, in this review, we include only the family, community, and policy levels.

### 3.10 Family Level

At the family level, stigma and cultural norms are the leading contributors to *pasung*. Family members often cite the inability to manage the behavior of a schizophrenia patient as a reason for resorting to physical restraint. The studies suggest that families view *pasung* as a temporary solution until other options become available.

### 3.11 Family Stigma and Protective Measures

Families play a significant role in the decision-making process regarding *pasung*. The desire to protect the family’s honor and social reputation can compel families to resort to restraint despite the negative consequences for the patient [12]. Schizophrenia patients often display aggressive and violent behavior, as reported in one of the studies, where patients with schizophrenia often wandered outside and damaged the surrounding furniture, neighbors’ environment/garden, stole food, threw things, and broke glass. In these circumstances, families often feel insecure and helpless. Thus, shackling became the practical solution out of fear for the security of the patients and the community [7-9, 11-13].

Additionally, economic stress can prevent families from seeking formal mental health care and see shackling as the solution for the family members with schizophrenia. In a study, based on family’s employment status, patients with unemployed and informal employment families were more likely to get *pasung* than patients with formal employment families [7]. In another study, families stated they cannot afford the mental healthcare costs. In some cases, they could not afford transportation costs even when they had health insurance [8]. Patients who had never been hospitalized before the implementation of the new health insurance system cited financial constraints as the main reason for not seeking treatment in the past [9].

Furthermore, the shame associated with having a relative with schizophrenia often leads to restraint as a way to hide them from public view. At times, patients expressed a willingness to consult a doctor or be transferred to a mental hospital, but their families often disregarded these requests for various reasons [8, 12]. Families who felt socially isolated due to having a member with a mental disorder believed it was necessary to bring their loved one to a psychiatric hospital discreetly to avoid drawing attention from others [11]. Therefore, family stigma plays a significant role in the decision to physically restrain individuals with schizophrenia.

### 3.12 Community Level

Community-level factors include a lack of resources and social isolation. In rural areas, the absence of mental health professionals, inadequate mental health education, and a general fear of the mentally ill lead communities to perpetuate the practice of *pasung* [8, 11, 12]. The community’s cultural beliefs about mental illness often reinforce stigma, making it difficult for families to seek alternative care.

### 3.13 Lack of Mental Health Resources and Education

The lack of mental health infrastructure and services in rural areas significantly influences the persistence of *pasung*. In these areas, families are often left with no other option but to rely on physical restraint to manage schizophrenia symptoms [7, 8, 13]. In the literature, families cited that access to mental health services in rural areas was severely limited. A lack of trust in the system was evident, and treated patients frequently experienced relapses. In contrast, mental health services in urban areas were comparatively better organized, offering both medical care and psychological counseling aimed at behavioral modification. Due to limited access, families often turned to alternative treatments that were more readily available [8]. Furthermore, the inadequate availability of mental health treatment within the community contributed to the practice of shackling individuals with mental disorders [11].

The absence of trained professionals limits the ability of communities to address mental health issues appropriately. The 2017 Ministry of Health Regulation designates Puskesmas (Indonesia’s Primary healthcare center) as key to mental healthcare, though they lack dedicated mental health services. They record mental health data and refer cases to sub-district Puskesmas. However, interviews revealed that doctors and nurses had no specialized mental health training and did not conduct outreach. Instead, sub-district welfare workers filled this gap. Mental illness cases were treated with medication, while severe cases were referred to a district urban Puskesmas, 20 km away. There are a limited number of hospitals that provide mental health services, each filled with 1 to 2 psychiatrists and 1 to 2 nurses. Unfortunately, none of these hospitals, as cited in the literature, provides an inpatient facility for mental health cases [10].

It is not only the lack of infrastructure that contributes to the issue, but also the community’s perceptions and attitudes toward schizophrenia, which influence access to care and treatment-seeking behavior. Families often sought alternative treatments for individuals with mental illness, turning to dukun (shamans) or Islamic religious leaders for healing practices such as rukiyah, a common Islamic method in Indonesia. Many participants in the study prioritized alternative treatments over medical care, often attempting them first. Additionally, the denial of mental illness within families led to avoidance of hospital treatment in favor of traditional methods. Some believed that since mental illness was caused by external forces, it should be treated through spiritual or traditional means [12].

Limited community awareness of schizophrenia and proper patient care contributed to widespread misconceptions. Many mistakenly viewed stopping aggressive behaviors and obedience to parents as signs of recovery, even when the patient remained in *pasung* (physical restraint) [8].

Another issue arises as some participants in Subu *et al.* (2023) reported that families of individuals with mental illness faced social isolation within their communities. Others noted that people avoided interacting with them or refused to communicate with them. One participant specifically described the different forms of isolation these families experienced. Participants described how community members would speak negatively about them but remain silent in their presence. They also noted that socioeconomic status influenced social support: wealthier individuals received more visits and care compared to poorer families, who were often avoided. As a result, some family members withdrew from social activities they once participated in, choosing to stay at home to avoid gossip and judgment. While a few individuals showed respect, the community's prevailing response was exclusion. To cope, some participants preferred to remain silent rather than confront negative remarks. This statement reflects the social stigma and isolation experienced by families of individuals with mental illness [12].

### 3.14 Policy Level

At the policy level, several articles highlight the role of government and mental health programs in reducing *pasung*. However, policy efforts have been insufficiently implemented in remote areas. National movements, such as “Indonesia Free *Pasung*”, aim to eradicate the practice, but local implementation and compliance are inconsistent [10]. Furthermore, existing policies often lack sufficient support for families in rural regions, where mental health resources are scarce.

### 3.15 Policy Gaps and Implementation Challenges

Over the past few decades, Indonesia has introduced a number of laws and initiatives aimed at improving mental health care and eliminating harmful practices such as *pasung*—the physical restraint or confinement of individuals with mental illness. One of the more ambitious programs was the *Stop Pasung Movement* in 2017, which set a target to make the country *pasung*-free by 2019 (later extended to 2023). As part of this initiative, a Social Rehabilitation Program was also launched to support the reintegration of individuals who had previously been subjected to restraint back into society [5].

Despite these national efforts, implementation has faced numerous setbacks, especially in rural areas. For example, the 2018 West Java Mental Health Regulation revealed substantial gaps in the availability and quality of services. Only a small percentage of primary health care facilities, around 20%, were able to provide mental health support. In addition, there was a shortage of trained professionals, essential medications, and necessary infrastructure. Access to psychiatric services was also limited by geographic remoteness and the lack of emergency mental health care, leaving tertiary hospitals to carry the burden of treating severe cases [5, 9, 10].

On a local level, the Aceh Free *Pasung* Movement stands out as a promising example of government intervention. Under this initiative, health insurance premiums are covered by the state, which theoretically removes financial barriers to care. However, a key issue remains unresolved: the cost of transportation. For many poor families living in isolated regions, traveling to a hospital or mental health center is financially unfeasible. Since transport is not included in the health or social insurance coverage, this often becomes a decisive barrier to accessing timely treatment [9].

Another local project also shows a positive change towards *pasung*. Another notable local initiative is the Sukabumi Project, which stands out for its multi-level coordination across all tiers of government. For the first time, the program involves officials ranging from village heads to sub-district and district authorities, in collaboration with the Ministries of Social Affairs and Health. The village head plays a central role by actively promoting the initiative within the community and overseeing its implementation. Additional support is provided by social welfare officers at the sub-district level (*Tenaga Kesejahteraan Sosial Kecamatan* or TKSK) and local community social workers (*Pekerja Sosial Masyarakat* or PSM), who facilitate and support ongoing service delivery [13].

What sets this project apart is its holistic approach to rehabilitation. The program focuses not only on individuals with mental illness but also offers support to their families. Rehabilitation efforts emphasize the development of practical life skills, enabling individuals to eventually reintegrate into society as contributing members. The reintegration process involves two critical stages: therapeutic and skill-building efforts conducted within the rehabilitation center, and community-based education designed to foster inclusion, reduce stigma, and promote a deeper understanding of mental illness and recovery.

An essential part of the program's success lies in changing community perceptions. Families and local residents are encouraged to recognize that, with appropriate care and support, individuals recovering from mental illness can once again become productive members of both the family and the broader community. In the context of Asian cultures, where familial bonds hold deep significance, this approach aligns well with the general desire of families to see their loved ones return home. It has already been noted that when individuals with mental illness go missing or become homeless, their families often make proactive efforts—by reaching out to social services, boarding houses, and hospitals, or relying on informal networks—to locate and bring them back.

A further achievement of the Sukabumi Project is the decentralization of access to essential medication. Previously, psychotropic drugs could only be prescribed and obtained from provincial hospitals, which are frequently located far from rural areas. Now, local health workers are authorized to order and manage these medications, which are stored at district-level *puskesmas* (community health centers). This development significantly reduces the time, cost, and logistical barriers that previously hindered access to psychiatric medication, marking an important step forward in local mental health care provision.

Despite the government’s clear intention to eliminate *pasung*, several underlying problems continue to undermine these efforts. Poor families, particularly in remote areas, often lack the financial means to seek medical care. Additionally, stigma within communities and even among family members towards mental illness—especially conditions like schizophrenia—remains widespread. A limited understanding of psychiatric disorders further contributes to the problem, leading some families to view *pasung* as the only viable option for managing a mentally ill relative. Without addressing these deeper social and economic factors, the complete eradication of *pasung* remains a challenging goal.

### 3.16 Consequences of *Pasung*

The consequences of *pasung* are both psychological and physical. The social stigma associated with *pasung* also causes psychosocial distress and further isolation from the community. Our findings revealed that individuals with mental illness often experienced significant social isolation within their communities. Many participants perceived this exclusion as stemming from the belief that these individuals were no longer viewed as productive members of society. Furthermore, participants noted that stigma extended beyond the patients themselves, affecting their families as well. Community members were often reluctant to engage or communicate with relatives of individuals living with mental illness. This reflects a broader pattern of social distancing and disconnection between these families and the wider community, as observed in the context of West Sumatra [12].

At the family level, guilt, anger, and mental distress are common among caregivers who feel trapped between societal expectations and the challenges of managing a schizophrenia patient [8, 11]. In one of our studies, twenty-one former *pasung* patients (35.6%) presented with notable muscle atrophy in their legs or arms upon admission to the hospital following their release from physical restraint. Nearly all individuals with lower limb atrophy experienced impaired mobility, with approximately half being unable to walk altogether. All affected patients required physical rehabilitation as part of their inpatient care [9].

### 3.17 Interventions and Policies

The Aceh Free *Pasung* and Sukabumi Project have played pivotal roles in reducing the use of *pasung* through policy implementation and community-based interventions. These programs aim to educate communities and provide alternative mental health care options. To date, only a limited number of studies have documented the local government’s efforts to reduce the practice of *pasung*. Nonetheless, it is important to acknowledge that over the past few decades, the Indonesian government has made considerable progress in formulating policies aimed at protecting the human rights of individuals with mental illness. One such initiative includes the integration of mental health services into the primary health care system. Currently, although with limited resources, mental health care is available at the community level through *Puskesmas* (community health centers), which operate in most sub-districts across the country [10, 12]. Evidence from one of the reviewed studies indicates a growing awareness and acceptance of formal treatment among both families and health care providers. Approximately two-thirds of patients previously subjected to *pasung* were brought in either by family members or referred by community mental health nurses based at primary care facilities. This suggests a shift in public and professional attitudes toward seeking proper psychiatric care. The remaining cases were identified through active outreach by hospital staff as part of the *Free Pasung* program, reflecting a stronger institutional commitment to case-finding and early intervention—an approach that was far less common in the past [9].

## 4 DISCUSSION

This systematic review underscores the continued use of *pasung* (physical restraint) among individuals with schizophrenia in Indonesia, despite nationwide initiatives aimed at its eradication. The practice is sustained by a complex interaction of factors operating at the familial, community, and policy levels, as framed by the Socio-Ecological Model (SEM). Although comprehensive national data remain limited due to inconsistent surveillance mechanisms, qualitative evidence indicates that *pasung* is particularly prevalent in rural and marginalized areas. Its impact is profound, often resulting in physical harm, emotional distress, and the exacerbation of psychiatric symptoms. Although Indonesia’s movement to free *Pasung* has achieved measurable progress, its overall effectiveness remains hindered by persistent structural and socio-cultural challenges. The human rights dimension of pasung must be explicitly situated within the international legal framework, particularly the United Nations Convention on the Rights of Persons with Disabilities (CRPD), to which Indonesia has been a signatory since 2007 and ratified through Law No. 19/2011. Article 13 of the CRPD asserts that persons with disabilities shall enjoy the right to liberty and security of person, and must not be deprived of their liberty unlawfully or arbitrarily. Additionally, Article 19 emphasizes the right to live independently and be included in the community, requiring states to provide access to community-based support services. The continued practice of pasung—physical restraint and confinement without due medical or legal oversight—constitutes a clear violation of these rights. Incorporating the CRPD into the national policy discourse on mental health would not only reinforce the moral and legal imperative to eliminate pasung but also hold the government accountable to its international obligations. This alignment is crucial to transition from punitive to rights-based approaches in mental health care [13].

The role of families and communities in sustaining *pasung* practices is deeply embedded in a complex matrix of cultural beliefs, limited support systems, and unrelenting caregiver stress. Findings from this review suggest that while families often act out of fear and desperation, their actions are shaped by an environment where formal mental health support is scarce or entirely absent [8, 10]. In such circumstances, *pasung* becomes not only a method of control but a reflection of structural neglect. Many caregivers, particularly in rural areas, face the overwhelming task of managing individuals with untreated psychosis without adequate knowledge, financial resources, or professional guidance [8, 9, 12].

Community attitudes further reinforce the normalization of restraint. In some regions, *pasung* is not condemned but quietly accepted—sometimes even conducted by community leaders or traditional healers [9, 10]. This social validation contributes to a cycle where stigma, shame, and isolation become part of the family's reality. Similar observations have been reported outside Indonesia; in countries like Nigeria and Ghana, spiritual explanations for mental illness continue to justify confinement in prayer camps or homes in lieu of psychiatric treatment. Such patterns suggest widespread failure to address mental illness not only through clinical care but also through public education and cultural dialogue [15].

To break this cycle, it is not enough to free individuals from physical restraint. Programs must address the underlying social structures and equip families with the tools they need to choose care over confinement. Empowering caregivers with culturally relevant mental health literacy, strengthening community-based support networks, and involving trusted traditional leaders in non-coercive models of care are essential steps forward. Without these, *pasung* may continue to be viewed not as a last resort, but as the only one.

One potential solution that can be proposed is the provision of structured training programs for traditional and spiritual healers. Traditional and spiritual healers may continue to play a dominant role in schizophrenia management across Indonesia and other Low- and Middle-Income Countries (LMICs). In many communities, especially in Indonesia and in another study in Egypt, traditional and spiritual healers are the first—and sometimes only—source of mental health support. In one study conducted in Egypt, when comparing patients’ experiences with traditional healers and psychiatrists, several participants reported a preference for traditional healing practices. The most cited reason was the greater acceptance of traditional healing within their communities. Affordability was another significant factor in choosing traditional healers due to lower costs, and the rest citing easier accessibility compared to formal psychiatric services. Notably, most who sought help from traditional healers believed that traditional treatment was more affordable than psychiatric care [16]. This reliance on traditional explanations and treatment delays timely diagnosis and access to psychiatric care.

However, integrating traditional healers into the formal mental health system has shown promising potential. A recent study conducted in Ghana by Yaro *et al.* highlights the positive impact of mental health literacy training for traditional and spiritual healers, particularly when supported by primary healthcare providers. The participants reported that the training significantly enhanced their understanding of mental health conditions and increased their awareness of the provisions outlined in Ghana’s Mental Health Act. Importantly, the training emphasized the human rights of individuals with mental illnesses, especially the need to treat both patients and their families with dignity and respect [17].

Among the key components of the training was a strong orientation on human rights, including the prohibition of chaining and shackling—a practice historically used to manage aggressive behavior. Although past studies often attribute the use of physical restraints to patients’ aggression, this study found that across all three regions examined in Ghana, neither patients nor their caregivers reported the use of chains or shackles, even in cases involving aggressive behavior. This suggests that the training effectively discouraged harmful practices by equipping healers with more humane approaches [18, 19].

Traditional and spiritual healers also shared alternative strategies they employ to manage aggressive patients. These include the use of calming herbal preparations, such as aromatic herbs applied near the nose to induce sleep, as well as special herbal baths. Some healers reported administering chlorpromazine, purchased from local drugstores, as part of their treatment regimen.

The study also revealed that the training fostered stronger collaboration between traditional healers and the formal health system, including hospitals and clinics. Health professionals, including Community Psychiatric Nurses (CPNs), now regularly visit shrines and healing camps to screen individuals for mental health conditions and administer biomedical treatments. Traditional healers also frequently contact CPNs for support in managing aggressive patients, allowing healthcare workers to administer calming injections and thereby avoid the need for physical restraints.

Overall, this collaboration has shown promising results. Traditional healers observed that combining their traditional practices with psychotropic medications from healthcare facilities accelerated patient recovery and helped prevent relapse. Furthermore, sustained use of these medications after discharge has contributed to a reduction in patient readmissions, easing overcrowding in traditional healing centers. Given Indonesia's deeply rooted religious and cultural values, many individuals seek help from these healers for mental health concerns. Therefore, such collaborative models could be adapted in Indonesia to reduce *pasung* and promote culturally sensitive care. By equipping them with basic mental health literacy, human rights awareness, and referral protocols, traditional and spiritual healers can play a more constructive role in the broader mental healthcare system. This approach not only respects cultural practices but also bridges the gap between traditional healing and modern psychiatric services, potentially improving access, reducing stigma, and enhancing the quality of care.

From a policy standpoint, while Indonesia’s efforts to end *pasung* represent a progressive shift toward deinstitutionalization and rights-based care, its implementation has been uneven. Several studies have revealed that the movement’s reach is limited by poor intersectoral coordination, inadequate follow-up, and lack of accountability at the local government level. In this context, *pasung* remains a fallback solution for families, tolerated as a normative practice rather than a violation of human rights. Collectively, these findings reflect a deep disconnect between policy intentions and ground-level realities. The Socio-Ecological Model (SEM) provides a useful framework for understanding this issue holistically: *pasung* is not only an individual or family choice but is also a reflection of broader community norms, healthcare system gaps, and policy enforcement failures.

Indonesia’s efforts to eradicate *pasung* have been supported by a succession of policies—from the 1977 Home Affairs decree to the 2010 Free *Pasung* Program to the Mental Health Act of 2014—but these measures have yielded only modest reductions in restraint practices. Ambiguous directives and overlapping mandates among ministries, provincial governments, and community actors have generated confusion over who is responsible for case identification, release, and rehabilitation, while incomplete decentralisation has left many districts under-resourced and ill-prepared to operationalize national goals [5].

In contrast, China’s 686 Program demonstrates how a clear, centrally coordinated policy can drive large-scale reductions in community restraint. Launched in 2005, the program “unlocked” patients held in family-imposed restraints and provided continuous, community-based follow-up care, resulting in sustained improvements in patient well-being and family burden. Its success has been attributed to well-defined stakeholder roles—linking provincial psychiatric hospitals, district health clinics, and community health workers in an integrated care pathway—and to robust data-monitoring systems that track releases, service uptake, and patient outcomes [20].

Similarly, Australia’s mental health reforms have emphasized comprehensive community care, backed by systematic evaluation. A recent review of Australian community-based mental health programs found that multidisciplinary therapeutic and case-management models significantly reduce psychiatric symptoms and improve psychosocial functioning for people with severe mental illness. Crucially, these programs are underpinned by national frameworks—such as the National Mental Health Strategy and successive Mental Health Plans—that specify service standards, workforce competencies, and funding mechanisms, enabling consistent implementation across states and territories [21].

England’s legislative approach further illustrates the value of clear policy architecture. The Mental Health Act reforms and associated community mental health transformation plans have established stringent criteria for detention, mandated patient choice and tribunal oversight, and set access and waiting-time standards for community services (Garratt 2024). These measures are supported by detailed operational guidance and accountability mechanisms within the NHS, ensuring that service providers at all levels understand their roles and responsibilities.

Meanwhile, in Indonesia, the Aceh Free *Pasung* program, launched in early 2010 by the Aceh provincial government, represents a landmark local initiative to eliminate the physical restraint of people with severe mental illness. Under the leadership of Governor Irwandi Yusuf, a dedicated ward was established at the Banda Aceh Mental Hospital, and a multidisciplinary “Free *Pasung*” team was formed to identify, release, and admit restrained individuals for comprehensive psychiatric and rehabilitative care. This effort was underpinned by the simultaneous introduction of the Aceh Health Insurance scheme (Jaminan Kesehatan Aceh), which, alongside the national Jamkesmas program, ensured that ex-*pasung* patients could access inpatient treatment without out-of-pocket costs, apart from transport. Strengths of the Aceh Free *Pasung* program include its clear political commitment and resource allocation at the provincial level, enabling rapid construction of infrastructure and staffing of a specialized treatment ward. The program’s active case-finding approach—through which nearly one third of patients were directly located and brought in by the Free *Pasung* team—demonstrates effective outreach and community engagement. Moreover, by integrating mental health service development with health-insurance reforms, Aceh addressed a key barrier—treatment affordability—thus aligning with WHO recommendations for universal health coverage in mental health.

Weaknesses remain in the program’s limited focus on acute hospital care, with insufficient mechanisms for long-term community-based follow-up, social reintegration, and support for families caring for individuals post-discharge. The cross-sectional study design of Puteh *et al.* captures demographic and clinical profiles—predominantly male, middle-aged, long-standing schizophrenia cases restrained for a mean of four years—but does not assess outcomes beyond hospital admission, nor the sustainability of freedom from restraint in community settings. Comparison with the national Free *Pasung* policy reveals that Aceh’s program benefits from more coherent local leadership and dedicated funding, whereas the national initiative (2010–2017) suffered from ambiguous directives, delayed operational guidelines, and fragmented decentralization that left many districts under-resourced and without clear implementation protocols. While both programs share the goal of eradicating *pasung*, Aceh’s model demonstrates the importance of coupling policy with concrete service-delivery structures and financing mechanisms—lessons the national government has sought to adopt through its 2017 Ministerial Decree No. 54 and subsequent reforms [9].

Nevertheless, the study examining the mental health policy framework still contains several shortcomings, particularly in its practical implementation. For instance, Indonesia’s policy framework for eradicating *Pasung* is extensive on paper but fragmented in practice. Although national-level regulations such as the Mental Health Act of 2014 and the Free *Pasung* Program exist, their implementation has been hampered by inconsistent messaging and unclear delineation of roles among policy actors. Stakeholders at every level receive differing directives, leading to confusion over who is responsible for case identification, release, and follow‐up care, and undermining accountability for outcomes.

A second major impediment is the incomplete decentralisation of health policy and service delivery. While decentralization reforms were intended to empower provincial and district governments to tailor mental health responses, many local authorities struggle with dual burdens: chronic under-resourcing and competing public‐health priorities. As a result, mental health remains a low priority, with insufficient budgetary commitment and limited local capacity to translate national goals into effective community‐level interventions.

Compounding these structural challenges is the absence of detailed operational guidance for those on the front lines. The Free *Pasung* Program was launched in 2010, yet no technical manual or stakeholder‐specific protocol was issued until Ministerial Decree No. 54 in 2017—seven years later. During this period, community health workers and local NGOs lacked clear job descriptions and standard operating procedures, resulting in ad hoc practices that failed to integrate medical, social, and legal responses to *Pasung* cases.

Legislative complexity further delays service expansion. The Mental Health Act of 2014 mandates at least five implementing regulations—one presidential and three ministerial—to operationalise comprehensive care, but not all have been enacted. Moreover, existing policies remain heavily institution-focused, emphasizing curative and rehabilitative services in hospitals with minimal attention to preventive, community-based approaches. Provincial pilot programs are unevenly distributed, and few include proactive outreach or family‐support components.

Finally, gaps in financing, data systems, and primary‐care capacity constrain progress. Social security laws (No. 40/2004 and No. 24/2011) promise coverage for people with psychosocial disabilities, yet eligibility criteria and premium‐subsidy mechanisms are vague, leaving many *Pasung* victims uninsured. There is no centralized data‐monitoring system to track releases or recidivism, undermining evaluation and planning. At the primary‐care level, although Puskesmas are formally empowered to deliver mental health services, only about 20 percent actually do so—largely due to limited training, staffing, and medication supplies.

Indonesia faces a critical shortage of specialist mental health professionals: recent stakeholder interviews confirm that the country has only 0.31 psychiatrists per 100,000 population, a figure far below the levels required to deliver effective, rights-based care at scale [22]. This scarcity is particularly alarming given the WHO Comprehensive Mental Health Action Plan 2013–2020, which underscores the necessity of an adequate specialist workforce to oversee diagnosis, medication management, and the integration of psychosocial interventions. Without sufficient psychiatrists, primary care clinics and community programs lack the expert oversight needed to manage complex cases, creating a vacuum that families often fill by resorting to *pasung* [5]. Compounding the numerical shortfall is a profound geographical maldistribution: the vast majority of Indonesia’s psychiatrists are concentrated in urban centres on Java, leaving remote provinces and rural districts effectively bereft of specialist services [22]. In these underserved areas, community health workers and general practitioners—already overstretched—must provide care for severe mental disorders with little to no specialist support. This mismatch between need and capacity undermines early detection, continuity of care, and the development of community-based follow-up, all of which are essential to prevent relapse and to sustain freedom from restraint.

While international examples such as Ghana, Egypt, and Australia offer valuable insights, their applicability to the Indonesian context must be interpreted with caution. Differences in health system infrastructure, resource availability, governance, cultural beliefs about mental illness, and the role of traditional healers can significantly influence the success of such models. For instance, Australia benefits from a robust primary care network and long-standing mental health legislation, whereas Indonesia faces challenges in rural mental health coverage and fragmented policy implementation. Therefore, while global comparisons are informative, contextual adaptation is essential for relevance and sustainability within Indonesia’s unique socio-cultural and health system landscape. The countries included for comparison in this review—such as Ghana, China, Egypt, and Australia—were selected based on several criteria. First, these countries have documented practices or historical parallels to *pasung*, such as the use of physical restraint or confinement of individuals with mental illness in community settings. Second, they represent a spectrum of policy responses, from low- and middle-income contexts (*e.g.*, Ghana, China, Egypt) to high-income countries with established mental health systems (*e.g.*, Australia). This range allows for examining how different health system capacities and cultural frameworks affect the implementation of deinstitutionalization programs. In particular, similarities in socioeconomic constraints, resource limitations, and family-centered care models in countries like Ghana and China provide useful analogs for understanding the Indonesian context.

The practice of *pasung* exerts severe and enduring psychopathological effects among individuals with schizophrenia. Prolonged physical restraint and social isolation—as widely documented in Indonesian settings—are associated with the crystallization of psychotic symptoms, in which delusions and hallucinations become more fixed and treatment-resistant due to lack of therapeutic engagement and environmental stimulation. Additionally, negative symptoms such as social withdrawal, flattened affect, and cognitive impairment often worsen in the absence of psychosocial interaction. Families and communities commonly perceive *pasung* as a pragmatic response to difficult behaviors, but this deprivation of autonomy and therapeutic continuity amplifies internalized stigma, traumatic stress, depression, anxiety, and feelings of hopelessness—all of which diminish motivation for recovery and trust in mental health services. Such conditions significantly increase the risk of chronic illness trajectories, impaired functioning, and reduced responsiveness to later interventions [8].

The dearth of psychiatrists directly perpetuates the practice of *pasung*. Families caring for a relative with severe psychosis or other high-risk conditions often have no access to timely specialist assessment or emergency medication. In the absence of mobile crisis teams or community outreach by mental health professionals, families’ fears of aggression or self-harm, unmitigated by adequate treatment, lead them to confine loved ones in shackles or cages [5]. Thus, the psychiatrist shortage is not merely a workforce statistic but a fundamental driver of ongoing human rights violations against people with mental illness. Addressing this gap requires a multi-pronged strategy: first, expanding psychiatric training positions and offering rural-service incentives to redistribute specialists; second, leveraging telepsychiatry to connect remote clinics with urban expertise; and third, formally integrating psychiatrists into task-sharing models that empower nurses and community health workers under specialist supervision [22]. Such measures, combined with sustained policy commitment and dedicated funding, are essential to build a resilient mental health system capable of finally ending *pasung* in Indonesia.

Although the *Free Pasung* initiative has been implemented in several regions, empirical evaluation of its long-term outcomes also remains limited. A two-year follow-up study in Central Java involving 114 individuals with severe mental illness who had been unlocked and treated at Soerojo Mental Hospital found that 24 % had been re-locked, and only 44 % adhered regularly to medication at follow-up. This highlights concerns about recidivism and weak continuity of care [6].

Indonesia has yet to adopt a comprehensive task-shifting approach for schizophrenia care, despite clear global precedents. In Indonesia, mental health cadres exist in theory, but their roles remain ill-defined and underutilized; detailed exploration of their implementation is scarce. The WHO explicitly endorses task-shifting—training non-specialists to deliver basic mental health interventions—to address workforce shortages. Yet, Indonesia’s national mental health plans have not translated this into large-scale Community Health Worker (CHW) programmes for severe mental illness [23]. By contrast, South Asia offers robust examples: India’s Accredited Social Health Activists (ASHAs) have been trained and incentivised to support people with severe mental illness, demonstrating feasibility and effectiveness in outreach, adherence monitoring, and psychoeducation [24]. Similarly, the Maanasi Project in rural Karnataka trained CHWs to screen, educate, and deliver therapies—including for schizophrenia—with ongoing specialist supervision, yielding improved access and continuity of care [25]. In sub-Saharan Africa, task-sharing models involving village-based health workers have successfully integrated mental health into primary care, reducing reliance on scarce psychiatrists [26, 27]. A second critical barrier is treatment non-adherence driven by stigma and supernatural beliefs. In Bali, 64 percent of family caregivers attributed schizophrenia to spiritual or magical causes, a perspective strongly linked to non-receipt of psychiatric treatment [28]. Among Indonesian patients themselves, beliefs in jinn possession, black magic, or divine punishment often lead families to consult dukun (folk healers) or Islamic religious healers before—and sometimes instead of—psychiatric services; these patterns delay biomedical care and perpetuate restraint practices [29]. Together, these workforce and cultural gaps form a vicious cycle sustaining *pasung*. Without CHWs to bridge the specialist shortage, families lack accessible, community-based alternatives to confinement; without culturally tailored psychoeducation to counter supernatural explanatory models, biomedical treatments remain underutilized. Addressing *pasung* thus requires formalised task-shifting—training and deploying CHWs for schizophrenia management—and integrating stigma-reduction strategies that respect religious frameworks while promoting evidence-based care [5].

## 5 LIMITATIONS

This review has several limitations. The evidence base is small, with only eight studies included, many of which had moderate methodological quality and relied heavily on indirect reporting from families rather than individuals with schizophrenia themselves. Most were qualitative or descriptive, limiting broader generalizability and causal interpretation. The lack of meta-analysis and the exclusion of non-English and non-Indonesian sources may also have restricted the scope of findings. Additionally, none of the included studies captured the direct voices or lived experiences of individuals with schizophrenia who had undergone *pasung*. This reflects a critical gap in the literature, likely due to ethical concerns, communication barriers, and stigma surrounding severe mental illness. Most studies prioritized perspectives of caregivers or professionals, limiting insight into the subjective and psychological impact of *pasung*. Future research should prioritize patient-centered approaches to ensure more inclusive and trauma-informed mental health responses.

## CONCLUSION

This review highlights that *pasung* remains a significant challenge in Indonesia, particularly for individuals living with schizophrenia, and is sustained by a complex interplay of stigma, inadequate services, cultural beliefs, and systemic gaps. While the included studies were diverse in design and scope, several common themes emerged, suggesting the need for multi-sectoral and community-based approaches to reduce reliance on physical restraint. Interventions such as public education, culturally adapted mental health care at the primary level, and improved psychosocial support appear promising but require further evaluation. Strengthening legal protections and increasing investment in mental health infrastructure may also contribute to long-term change. However, given the exploratory nature of this review and the limited number of high-quality studies, these conclusions should be interpreted with caution. Further research is essential to assess the effectiveness of existing policies and to understand how best to support families and communities in transitioning away from coercive practices. Ultimately, addressing *pasung* involves not only improving services but also promoting dignity, rights, and social inclusion for people with severe mental illness in Indonesia.

## Figures and Tables

**Table 1:** Key findings of included studies.

**No.**	**Title**	**Study Design**	**Citations**	**Main Objectives**	**Key Findings**
1.	Factors associated with *pasung* (physical restraint and confinement) of schizophrenia patients in Bogor regency, West Java Province, Indonesia, 2017.	Policy analysis	(Laila *et al.*, 2019) [7].	To analyze the development and implementation of Indonesia’s *Free Pasung* policy using the Walt and Gilson policy triangle framework, examining the policy context, content, actors, and processes involved.	• Most patients reside in rural areas. • *Pasung* was initiated by the father, mother, or elder siblings. • Patient’s aggressive or violent behavior, relapse, unemployment in the family, and negative attitude of the family were associated with *pasung* among patients with schizophrenia • Patients with aggressive behavior are more likely to be *Pasung* than those with non-aggressive behavior. • Among patients with aggression who were restrained (*pasung*), 77.7% of their *pasung* cases are due to patients' aggression. • Unemployment in the family is the biggest contributing factor to *pasung* at the population level. • If family unemployment were eliminated, the number of *pasung* cases in the entire population could decrease by 49.3%.
2.	Perceptions about *pasung* (physical restraint and confinement) of schizophrenia patients: a qualitative study among family members and other key stakeholders in Bogor regency, West Java Province, Indonesia 2017.	Cross-sectional study	(Laila *et al.*, 2018) [8].	To identify factors associated with the use of *pasung* among schizophrenia patients in Bogor Regency, Indonesia, using quantitative analysis of secondary health surveillance data.	• According to family members, neighbors, and community leaders, the primary reason for implementing *pasung* was to ensure the safety of both the patient and those around them. Many patients exhibited aggressive behaviors, including physical violence toward their relatives and neighbors. In such situations, family members felt vulnerable and uncertain about how to manage the situation. • Family and community members viewed *pasung* as the only viable option to protect both the patient and others from aggressive or destructive behavior. It was considered a practical solution for individuals with mental illness and had become a socially accepted norm. In some cases, patients expressed a desire to see a doctor or be transferred to a mental hospital, but their families often disregarded these requests for various reasons. • Families struggled to afford mental healthcare costs, and even those with health insurance often found transportation expenses prohibitive. Additionally, they faced financial difficulties in providing food for patients with excessive eating habits. As a result, many families were unable to meet even their own basic needs. • Mental health services in rural areas were severely limited, with low availability and accessibility. There was a lack of trust in the system, as treated patients often experienced relapses. In contrast, urban areas had better-coordinated mental health services, including medical care and psychological counseling aimed at behavioral change. Due to these challenges, families frequently turned to alternative treatments that were more readily accessible. • The community had limited awareness of schizophrenia and how to care for individuals with the condition. In contrast, misconceptions and misunderstandings were widespread. Behaviors such as stopping aggression and obeying parents were mistakenly seen as signs of recovery, even when the patient was restrained in *pasung*.
3.	Aceh free *pasung*: releasing the mentally ill from physical restraint.	Qualitative study (focus group discussions and interviews).	(Puteh *et al.*, 2011) [9].	To explore the perceptions and experiences of family members and other stakeholders regarding the use of *pasung* for schizophrenia patients in Bogor Regency.	• Of the 59 patients, the primary concern for 47 patients was their aggressive behavior. In the other 12 cases, the focus was on the patient's safety and well-being due to wandering, while a few cases cited various “special reasons.” For instance, some patients were restrained shortly after the launch of the Aceh Free *Pasung* program, with the hope that “someone from the government would come, release them, and take them to the hospital for treatment without any cost.” • In most cases (86.4%), the decision to use *pasung* was made by the patient's family. In the remaining cases (13.6%), it was community leaders who decided to apply *pasung*. • Patients who had been in *pasung* for longer periods were less likely to have had previous psychiatric treatment, whereas those with shorter durations of *pasung* typically had received treatment at least once. • At the time of their release from *pasung* and admission to the hospital, 21 ex-*pasung* patients (35.6%) showed significant muscle atrophy in their legs or arms. Almost all of those with lower extremity atrophy experienced difficulty walking; about half were unable to walk at all, and all required physical therapy during their hospitalization. • Many of the respondents mentioned that, in addition to previous hospital treatment, they had also sought care from traditional or religious healers. About a quarter of the patients who had never been hospitalized before the introduction of the new health insurance schemes cited the inability to pay for hospital services as the reason they had not sought treatment earlier.
4.	Evaluating the Indonesian free *pasung* movement: understanding continuing use of restraint of the mentally ill in rural Java.	Descriptive case study	(Hunt *et al.*, 2023) [10].	To describe the implementation and outcomes of the “Aceh Free Pasung” program, which aimed to release and rehabilitate mentally ill individuals from physical restraint in Aceh Province.	• The Puskesmas system is central to mental health care but lacks specialized training and direct mental health services. While mental health data is recorded, no explicit mental health services are provided. • Mental health care in Winong and nearby areas is limited, with referrals to other facilities required for more extensive care (sub-district Puskesmas). • Secondary and tertiary care is also limited by insurance coverage and transportation costs, with significant gaps in access for low-income individuals, including many with mental illness. Many Indonesians, especially those with mental illness, are not registered in the national insurance scheme, making access to care more difficult for low-income groups. • Continuity of care after discharge is problematic due to logistical issues, including long travel distances for medication. After discharge, patients ideally continue their treatment through outpatient primary care, but many patients fail to reach their intended facilities, and those who do often find the required medications unavailable, forcing them to travel long distances to obtain prescriptions. • There is a significant gap in government-provided day care, rehabilitative, and residential care services in Winong, which has led to the emergence of a private residential care facility. The 2017 Ministry of Health Regulation emphasizes the importance of family support for individuals with mental illness to assist with care, rehabilitation, and reintegration into the community post-hospitalization. Rehabilitative services are meant to enhance patients’ social and vocational skills for reintegration. However, no day care or rehabilitative services, as outlined in the 2017 regulation, were found in the mental healthcare mapping in the area. • When family support is absent, the government assumes responsibility for the care of individuals with mental illness, as per the Mental Health Legislation of 2014. The 2017 Ministry of Health Regulation mandates government-run residential care facilities, but no such facilities were available in Winong (Indonesia’s district) or surrounding areas during the 2015–2016 interviews. A private initiative, run by an individual, operated a residential care facility in Winong village to address the gap.
5.	Indonesia free from *pasung*: a policy analysis.	Mixed-methods evaluation study.	(Hidayat *et al.*, 2023) [5].	To evaluate the effectiveness and challenges of the Indonesia *Free Pasung* movement by exploring continued practices of restraint in rural Java and the perspectives of mental health stakeholders.	• **1966–1998 (New Order Era)**: The government focused on institutionalized psychiatric care, increasing the number of psychiatric hospitals. • **1977 Ministerial Decree**: Encouraged public awareness against ***Pasung*** and instructed local leaders to address mental health issues in communities • **1992 Health Law**: Shifted focus toward community-based mental health support, acknowledging the connection between mental health, poverty, and unemployment. • **1999 Human Rights Act**: Recognized freedom from torture, including seclusion and restraint of mentally ill individuals, as a fundamental human right. • **2010 ‘Towards Indonesia Free of *Pasung’* Campaign**: Launched to eliminate ***Pasung***, backed by advocacy groups and media; Emphasized government responsibility in providing mental health services; Encouraged community health centers (Puskesmas) to be the first point of contact for treatment. • **2011 Social Security Law**: Provided financial support for low-income individuals, improving healthcare accessibility. • **2014 Mental Health Act**: Criminalized ***Pasung***, though enforcement remained unclear. • **2016 Disability Rights Law**: Stressed protection against torture and mistreatment for individuals with disabilities, including mental illness. • **2017 Stop *Pasung* Movement**: Aimed to make Indonesia ***Pasung*-free** by 2019 (later revised to 2023); Introduced a **Social Rehabilitation Program** to reintegrate individuals previously subjected to ***Pasung***. • **2017 Ministerial Decree on Stop *Pasung***: Outlined the mental health service system, emphasizing community-based treatment and structured referral pathways. • **2018 West Java Mental Health Regulation**: Highlighted major gaps in mental health services: Only **20% of primary healthcare facilities** provided mental health services; Limited resources, medications, and trained personnel; Access to psychiatric care was hindered by **remoteness and lack of emergency services;** Tertiary hospitals have become the main providers for severe cases.
6.	Family stigma correlation with shackling in schizophrenia patients in the Psychiatric hospital of Bali province.	Cross-sectional correlational study.	(Jayanti & Dharmaw an, 2017) [11].	To assess the correlation between family stigma and the use of *pasung* among schizophrenia patients at a psychiatric hospital in Bali Province.	• Lack of knowledge about mental disorders leads to poor treatment quality. Many families hide mental illness and quietly take patients to psychiatric hospitals to avoid social judgment. • A strong relationship exists between family stigma and the practice of shackling schizophrenia patients. High willingness among families to shackle schizophrenia patients is driven by limited knowledge, lack of mental health facilities, and safety concerns. • Chronically ill schizophrenia patients are perceived as dangerous due to behaviors like violence, property destruction, and aggression.
7.	“Family stigma” among family members of people with mental illness in Indonesia: a grounded theory approach.	Grounded theory qualitative study.	(Subu *et al.*, 2023) [12].	To develop a theoretical understanding of how family stigma is constructed and experienced by family members of people with mental illness in Indonesia.	• Use of *pasung* is common in some areas of Indonesia for managing mental illness within families. • Some families believe mental illness is caused by spirit possession (jinn, demons, or devils). As a result, they seek alternative treatments from shamans (*dukun*) or Islamic religious leaders. • Some prefer to go for alternative or religious treatment, like Shamanic treatments, conducted by a *dukun* using traditional healing methods, or Islamic healing (Rukiyah): A method used by religious leaders to expel spirits. • Families of mentally ill individuals face community rejection and social isolation. People avoid communicating with them, reinforcing exclusion and stigma.
8.	Introducing recovery-oriented practice in Indonesia: the Sukabumi Project – an innovative mental health programme.	Program description and process evaluation.	(Stratford *et al.*, 2014) [14].	To describe and evaluate the Sukabumi Project, an innovative mental health program introducing recovery-oriented practices in Indonesia.	• Rehabilitation programs aim to develop essential life skills that help individuals reintegrate into society and become active members of their communities. A key aspect of fostering acceptance is demonstrating to families that, with proper treatment and support, individuals with mental illness can contribute positively to both their families and the broader community. • Most families ultimately wish for their loved ones with mental illness to return home and reintegrate into family life. In cases where individuals become homeless, families often reach out to social welfare services, boarding houses, psychiatric hospitals, or informal networks to locate and bring them back. • Many families resort to *pasung* (physical restraint) as a means of protecting both the individual and the community, especially in response to violent or disruptive behavior. However, this practice is largely driven by a lack of mental health education and limited access to affordable treatment, leaving families with no other options. • To address these challenges, a community-based rehabilitation system is crucial. Such a system would provide necessary support to individuals facing mental health struggles, including those subjected to *pasung* or those who have been displaced due to homelessness or institutionalization.

## Data Availability

The data supporting the findings of the article is available in the the FigShare at [https://figshare.com/], [DOI: 10.6084/m9.figshare.30564710].

## LIST OF ABBREVIATIONS

CASP: Critical Appraisal Skills Programme
CHW: Community Health Worker
CPN: Community Psychiatric Nurse
ICF: International Classification of Functioning, Disability and Health *(if mentioned, otherwise ignore)*
JBI: Joanna Briggs Institute
LMICs: Low- and Middle-Income Countries
NGO: Non-Governmental Organization
PRISMA: Preferred Reporting Items for Systematic Reviews and Meta-Analyses
SEM: Socio-Ecological Model
TKSK: *Tenaga Kesejahteraan Sosial Kecamatan*
WHO: World Health Organization

## STANDARDS OF REPORTING

PRISMA guidelines and methodology were followed.

## SUPPLEMENTARY MATERIAL

PRISMA checklist is available as supplementary material on the publisher’s website along with the published article.
