# Supplementary material for: From Chains to Care: Ending Pasung (Physical Restraint) Among Schizophrenia Patients in Indonesia, A Systematic Review
Source: Clin Pract Epidemiol Ment Health. 2025 Nov 12;21:e17450179414516. doi: 10.2174/0117450179414516251030074059 (PMC12895430; doi:10.2174/0117450179414516251030074059)
Supplement: Supplementary file 1 — PRISMA checklist is available as supplementary material on the publisher’s website along with the published article. [file CPEMH-21-E17450179414516_SD1.pdf]

Clin. Pract. Epidemiol. Ment. Health
ISSN: 1745-0179
DOI: 10.2174/0117450179414516251030074059, 2025, 21, e17450179414516
1
SUPPLEMENTARY MATERIAL
OPEN ACCESS

From Chains to Care: Ending *Pasung* (Physical Restraint) Among Schizophrenia Patients in Indonesia, A Systematic Review

Jesslyn Shi[1], Rama Mahardika Primanov[1], Rina Amelia[2,*] and Nanda Andini[1]

[1]*Undergraduate Program in Medicine, Faculty of Medicine, Universitas Sumatera Utara, Medan, Indonesia*
[2]*Department of Community Medicine, Faculty of Medicine, Universitas Sumatera Utara, Medan, Indonesia*

© 2025 The Author(s). Published by Bentham Open.
This is an open access article distributed under the terms of the Creative Commons Attribution 4.0 International Public License (CC-BY 4.0), a copy of which is available at: https://creativecommons.org/licenses/by/4.0/legalcode. This license permits unrestricted use, distribution, and reproduction in any medium, provided the original author and source are credited.

* Address correspondence to this author at the Department of Community Medicine, Faculty of Medicine, Universitas Sumatera Utara, Medan, Indonesia; Tel: 08116180352; E-mail: rina2@usu.ac.id

Cite as: Shi J, Primanov R, Amelia R, Andini N. From Chains to Care: Ending *Pasung* (Physical Restraint) Among Schizophrenia Patients in Indonesia, A Systematic Review. Clin Pract Epidemiol Ment Health, 2025; 21: e17450179414516. http://dx.doi.org/10.2174/0117450179414516251030074059

Received: May 27, 2025
Revised: September 3, 2025
Accepted: July 19, 2025
Published: November 12, 2025

Send Orders for Reprints to reprints@benthamscience.net

PRISMA 2020 Checklist

Section and Topic	Item #	Checklist item	Location where item is reported
**TITLE**			
Title	1	Identify the report as a systematic review.	Title section, page 1
**ABSTRACT**			
Abstract	2	See the PRISMA 2020 for Abstracts checklist.	Abstract section, page 1
**INTRODUCTION**			
Rationale	3	Describe the rationale for the review in the context of existing knowledge.	Introduction section, page 2 (last 3 sentences)
Objectives	4	Provide an explicit statement of the objective(s) or question(s) the review addresses.	Introduction section, page 2 (last sentence)
**METHODS**			
Eligibility criteria	5	Specify the inclusion and exclusion criteria for the review and how studies were grouped for the syntheses.	Sub-section: literature searching, page 2
Information sources	6	Specify all databases, registers, websites, organisations, reference lists and other sources searched or consulted to identify studies. Specify the date when each source was last searched or consulted.	Sub-section: literature searching, page 2
Search strategy	7	Present the full search strategies for all databases, registers and websites, including any filters and limits used.	Sub-section: literature searching, page 2
Selection process	8	Specify the methods used to decide whether a study met the inclusion criteria of the review, including how many reviewers screened each record and each report retrieved, whether they worked independently, and if applicable, details of automation tools used in the process.	Sub-section: screening process, page 2-3
Data collection process	9	Specify the methods used to collect data from reports, including how many reviewers collected data from each report, whether they worked independently, any processes for obtaining or confirming data from study investigators, and if applicable, details of automation tools used in the process.	Sub-section: screening process, page 2-3

Section and Topic	Item #	Checklist item	Location where item is reported
Data items	10a	List and define all outcomes for which data were sought. Specify whether all results that were compatible with each outcome domain in each study were sought (e.g. for all measures, time points, analyses), and if not, the methods used to decide which results to collect.	Sub-section: literature searching, page 2
	10b	List and define all other variables for which data were sought (e.g. participant and intervention characteristics, funding sources). Describe any assumptions made about any missing or unclear information.	Sub-section: literature searching, page 2
Study risk of bias assessment	11	Specify the methods used to assess risk of bias in the included studies, including details of the tool(s) used, how many reviewers assessed each study and whether they worked independently, and if applicable, details of automation tools used in the process.	Sub-section: quality assessment, page 3
Effect measures	12	Specify for each outcome the effect measure(s) (e.g. risk ratio, mean difference) used in the synthesis or presentation of results.	Sub-section: data extraction and analysis, page 3
Synthesis methods	13a	Describe the processes used to decide which studies were eligible for each synthesis (e.g. tabulating the study intervention characteristics and comparing against the planned groups for each synthesis (item #5)).	Sub-section: data extraction and analysis, page 3
	13b	Describe any methods required to prepare the data for presentation or synthesis, such as handling of missing summary statistics, or data conversions.	Sub-section: data extraction and analysis, page 3
	13c	Describe any methods used to tabulate or visually display results of individual studies and syntheses.	Sub-section: data extraction and analysis, page 3
	13d	Describe any methods used to synthesize results and provide a rationale for the choice(s). If meta-analysis was performed, describe the model(s), method(s) to identify the presence and extent of statistical heterogeneity, and software package(s) used.	Sub-section: data extraction and analysis, page 3
	13e	Describe any methods used to explore possible causes of heterogeneity among study results (e.g. subgroup analysis, meta-regression).	Sub-section: data extraction and analysis, page 3
	13f	Describe any sensitivity analyses conducted to assess robustness of the synthesized results.	Sub-section: quality assessment, page 3
Reporting bias assessment	14	Describe any methods used to assess risk of bias due to missing results in a synthesis (arising from reporting biases).	Sub-section: quality assessment, page 3
Certainty assessment	15	Describe any methods used to assess certainty (or confidence) in the body of evidence for an outcome.	Sub-section: quality assessment, page 3
**RESULTS**			
Study selection	16a	Describe the results of the search and selection process, from the number of records identified in the search to the number of studies included in the review, ideally using a flow diagram.	Figure 1 (PRISMA Flow Diagram), page 4
	16b	Cite studies that might appear to meet the inclusion criteria, but which were excluded, and explain why they were excluded.	Figure 1 (PRISMA Flow Diagram), page 4
Study characteristics	17	Cite each included study and present its characteristics.	Table 1 (Key findings of included studies), page 6
Risk of bias in studies	18	Present assessments of risk of bias for each included study.	Results-Data Characteristics, last sentence of each paragraph
Results of individual studies	19	For all outcomes, present, for each study: (a) summary statistics for each group (where appropriate) and (b) an effect estimate and its precision (e.g. confidence/credible interval), ideally using structured tables or plots.	Table 1 (Key findings of included studies), page 6
Results of syntheses	20a	For each synthesis, briefly summarise the characteristics and risk of bias among contributing studies.	Table 1 (Key findings of included studies), page 6
	20b	Present results of all statistical syntheses conducted. If meta-analysis was done, present for each the summary estimate and its precision (e.g. confidence/credible interval) and measures of statistical heterogeneity. If comparing groups, describe the direction of the effect.	Table 1 (Key findings of included studies), page 6
	20c	Present results of all investigations of possible causes of heterogeneity among study results.	Table 1 (Key findings of included studies), page 6
	20d	Present results of all sensitivity analyses conducted to assess the robustness of the synthesized results.	Table 1 (Key findings of included studies), page 6
Reporting biases	21	Present assessments of risk of bias due to missing results (arising from reporting biases) for each synthesis assessed.	Results-Data Characteristics, last sentence of each paragraph
Certainty of evidence	22	Present assessments of certainty (or confidence) in the body of evidence for each outcome assessed.	Table 1 (Key findings of included studies), page 6
**DISCUSSION**			
Discussion	23a	Provide a general interpretation of the results in the context of other evidence.	Discussion section, page 11-14
	23b	Discuss any limitations of the evidence included in the review.	Discussion section, page 14-15, last paragraph
	23c	Discuss any limitations of the review processes used.	Discussion section, page 14-15, last paragraph
	23d	Discuss implications of the results for practice, policy, and future research.	Conclusion section, page 14-15

Section and Topic	Item #	Checklist item	Location where item is reported
**OTHER INFORMATION**			
Registration and protocol	24a	Provide registration information for the review, including register name and registration number, or state that the review was not registered.	Methods section, first sentence, page 2
	24b	Indicate where the review protocol can be accessed, or state that a protocol was not prepared.	Methods section, first sentence, page 2
	24c	Describe and explain any amendments to information provided at registration or in the protocol.	Methods section, first sentence, page 2
Support	25	Describe sources of financial or non-financial support for the review, and the role of the funders or sponsors in the review.	Funding section, page 15
Competing interests	26	Declare any competing interests of review authors.	Conflict of interest section, page 15
Availability of data, code and other materials	27	Report which of the following are publicly available and where they can be found: template data collection forms; data extracted from included studies; data used for all analyses; analytic code; any other materials used in the review.	Data used for analyses, by request to author

From: Page MJ, McKenzie JE, Bossuyt PM, Boutron I, Hoffmann TC, Mulrow CD, et al. The PRISMA 2020 statement: an updated guideline for reporting systematic reviews. BMJ 2021;372:n71. doi: 10.1136/bmj.n71.
